# Impacts of invasion on a freshwater cleaning symbiosis

**DOI:** 10.1007/s00442-024-05600-4

**Published:** 2024-08-03

**Authors:** Spencer S. Bell, Philip McElmurray, Robert P. Creed, Bryan L. Brown

**Affiliations:** 1 Department of Biological Sciences, Virginia Tech, Blacksburg, VA 24061 USA; 2 Department of Biology, Appalachian State University, Boone, NC 28608 USA; 3 Present Address: Department of Anthropology, Washington University in St. Louis, St. Louis, MO 63130 USA

**Keywords:** Crayfish, Branchiobdellidans, Noncompetent hosts, Dispersal, Invasive species, Native symbionts

## Abstract

Organismal invasions have repeatedly been cited as a driving force behind the loss of biodiversity. Unlike many other impacts of invasion, the effect of invasion on native symbiont communities has received less attention. The introduction of invasive hosts presents a potential opportunity to native symbionts; invasive hosts could benefit native symbionts through providing a novel host environment that improves symbiont fitness relative to their fitness on native hosts. Alternatively, invasive hosts could noncompetent hosts for native symbionts, resulting in negative impacts on native symbiont abundance and diversity. Crayfish in the northern hemisphere host diverse assemblages of obligate annelid symbionts (P: Anellida, O: Branchiobdellida). Two invasive crayfish hosts in the genus *Faxonius* have been introduced and are interacting with the native crayfish hosts and their symbionts in three watersheds in western Virginia, USA. Previous studies suggest that the invasive host *F. cristavarius* is a less competent host for symbionts compared to native hosts in the genus *Cambarus*. We carried out an extensive survey in these watersheds to determine impacts of varying degrees of invasion on branchiobdellidan abundance and diversity. We also conducted a complementary host replacement experiment to investigate how increases in the relative abundance of invasive hosts contributes to observed patterns of symbiont abundance and diversity in the field. In our survey, as the proportion of invasive hosts at a site increased, branchiobdellidan abundance and diversity declined significantly. In the experiment, the worms dispersed onto both native and invasive hosts. As the percentage of noncompetent *F. cristavarius* hosts increased, the survival of branchiobdellidans declined. Both symbiont survival and opportunities for successful dispersal are reduced as this noncompetent invasive host progressively displaces native hosts, which imperils the integrity of native host-symbiont systems. Given that many native hosts accrue significant fitness benefits from their relationships with native symbionts, including hosts in our study system, losses of beneficial symbionts may produce a positive feedback loop that decreases invasion resistance of native species, exacerbates the effects of invasions, and presents a major conservation issue in invaded systems.

**Supplementary Information:**

The online version contains supplementary material available at 10.1007/s00442-024-05600-4.

## Introduction

Invasive species are widely recognized as one of the preeminent threats to biodiversity and ecosystem functioning worldwide and have impacts that extend beyond the biological into the economic and societal (Pimentel et al. 2005; Strayer and Dudgeon 2010; Vilà et al. 2011). Species invasions are a major contributor to global change that result in consequences that are both ecological and economic (Diagne et al. 2021; Didham et al. 2005; Hellmann et al. 2008; MacDougall and Turkington 2005). Increasing levels of global connectivity have increased the rates at which invasive species are introduced into new ecosystems, further elevating the threat posed by invaders (Crooks and Suarez 2006), and rates of invasions continue to increase across all taxonomic groups (Seebens et al. 2018).

Many effects of invasive species have been well-documented, including effects on biodiversity, impacts on ecosystem services, risks to human health, and severe economic consequences as a result of those impacts (Pyšek and Richardson 2010). Despite considerable progress in understanding invasions and their effects, one of the major shortcomings of invasive species research to-date is that the majority of work has focused on the effects of single target species in single-patch studies (Brown and Barney 2021; Pyšek and Richardson 2007, 2010). However, there is widespread recognition that a focus on the invaded community, rather than a specific focus on the invader, would be potentially revealing (Gallien and Carboni 2017; Hui and Richardson 2017; Shea and Chesson 2002). Viewing invasions in a broader community context has the potential to not only increase our understanding of invader success or failure, but also to better understand the effects of invaders that are transmitted through ecological communities through both direct and indirect interactions (Brown and Barney 2021; Pearson et al. 2018; Shea and Chesson 2002).

One facet of ecological communities that has largely been ignored by invasion science is the effect of invaders on native symbioses. This omission is not surprising since symbioses—particularly positive interactions like mutualism—have been vastly understudied in ecology as a whole, despite their importance to the maintenance of biodiversity (Dehling et al. 2022; Silknetter et al. 2020; Stachowicz 2001). Most plants and virtually all metazooans are involved in some type of symbiotic interaction, and consequently, nearly all invasions have the potential to influence symbioses that occur in the invaded community. When invasive species enter novel systems, they may interact not only with native hosts, but also with their symbionts. While there have been extensive studies of native and introduced symbiont effects on non-indigenous plant hosts (Klironomos 2002; Richardson et al. 2000b; Traveset and Richardson 2014), there have been far fewer studies of how invasive animal hosts may interact with native symbiont communities (Creed et al. 2022a, b; Keesing et al. 2009; Let et al. 2023). Most work on animal hosts focuses on interactions with parasites and pathogens and particularly on the enemy release hypothesis as it relates to parasites and pathogens left behind in an invader’s native range (Dunn and Hatcher 2015; Dunn et al. 2012). Results from these studies suggest that invasive species may benefit from the inability of their associated parasites to transfer to novel ecosystems. These invasive species may be lower quality hosts for native symbionts compared to their native hosts (Roy et al. 2011; Torchin et al. 2003).

From the perspective of a native symbiont, the introduction of an invasive host species represents a potential opportunity but the opportunity is highly contingent on the properties of the invader (Creed et al. 2022b). The newly introduced species may present a novel host environment that supports the expansion of native symbiont communities. Alternatively, an invasive host may offer an inferior environment compared to native hosts and negatively affect symbiont communities (Creed et al. 2022b). If an invasive host species is noncompetent with respect to native symbionts, colonization of the invasive host by native symbionts may result in degradation of the local symbiont community with invasive hosts acting as a sink habitat for native symbionts. This scenario could result in reduced symbiont loads on native hosts and the loss of fitness benefits that native hosts may derive from mutualisms with these symbionts (Creed et al. 2022a, b; Mastitsky et al. 2010; Mestre et al. 2015) and is similar to the *dilution effect* described for the effect of high densities of non-competent hosts on parasites (Civitello et al. 2015; Huang et al. 2016; Keesing et al. 2006). Native symbionts may also aid native hosts in resisting displacement by invasive species, or aid invasive species in spreading in novel environments (Creed et al. 2022b).

We investigated the effects of host invasion on native symbionts by studying the invasion of non-native crayfish and their effects on ectosymbiotic crayfish worms. The relationship between crayfish and branchiobdellidans is a context-dependent mutualism for several documented host-symbiont combinations—including the native hosts of this study—in which the symbionts’ foraging activity acts as a cleaning service, reducing host mortality and increasing host growth by up to 45% (Ames et al. 2015; Brown et al. 2012, 2002; Lee et al. 2009). The Mountain Lake region of western Virginia, USA, has experienced the introduction and subsequent spread of a number of invasive crayfish, including *Faxonius cristavarius*, *F. virilis*, and *Procambarus clarkii*. Previous work on the invasive crayfish *Faxonius cristavarius* (nee *Orconectes cristavarius*) suggests that they are poor hosts for native branchiobdellidan symbionts (Farrell et al. 2014). This species has spread throughout the region since its introduction in the 1930s and has become the dominant crayfish in many streams in the last few decades. We therefore hypothesized that the introduction and spread of the invasive crayfish *F. cristavarius* has resulted in negative impacts on the local symbiont community throughout this region. We further hypothesized that the mechanism behind these negative impacts is due to a version of the dilution effect where the colonization of non-competent invasive hosts by native symbionts results in high symbiont mortality (Creed et al. 2022a, b). Potentially magnifying the effects of increased mortality are Allee effects among branchiobdellidans (Creed and Brown 2018) so that reduced population size may also lead to decreases in per-capita reproductive rates (Creed and Brown 2018). Evidence of this phenomenon in the crayfish-branchiobdellidan system has already emerged in Europe where branchiobdellidans have declined in the presence of invasive species (Let et al. 2023). To study the impacts of invasive hosts on native symbionts, we conducted a large survey, as well as a mesocosm experiment to test potential mechanisms of negative effects of invaders on native symbionts.

## Methods

### Study system

The Mountain Lake region of Virginia spans three river drainages, the New, the James, and the Roanoke, and hosts a diverse assemblage of both crayfish and crayfish symbionts (Hobbs et al. 1967). The most conspicuous of these symbionts are the ectosymbiotic branchiobdellidans, also known as crayfish worms (Hobbs et al. 1967). These annelid worms are close relatives to leeches (P: Annelida, C: Clitellata, sC: Hirudinoidea, O: Branchiobdellida) that are obligate symbionts of crayfish and are dependent on crayfish hosts for both reproduction and dispersal, the latter only occurring through direct crayfish to crayfish contact for most studied species (Creed et al. 2015; Gelder 2010; Skelton et al. 2013; Young 1966). These worms feed on biofilms, protozoa, and metazoans, including other branchiobdellidans, that live on the crayfish exoskeleton and gills (Skelton et al. 2013). Branchiobdellidans vary widely in their abundances and diversity on native hosts. It is not unusual to find several species on a single host, and total branchiobdellidan abundances on a single host can be in the 100 s (Skelton et al. 2013; Vlach et al. 2017), though these patterns vary depending on host, particular species of branchiobdellidan, and environmental conditions (Skelton et al. 2013).

There can be considerable variation in the quality of crayfish as hosts for branchiobdellidans, and that quality is related to the size and species of the crayfish. Older and larger native *Cambarus* crayfish have significantly higher tolerance of worms than younger, smaller crayfish that actively control worm abundances through grooming behaviors (Farrell et al. 2014; Skelton et al. 2014; Thomas et al. 2016). This relationship between symbiont tolerance and host size is believed to be related to the molting periodicity of the crayfish host (Brown et al. 2012). Host molting reduces potential food sources for worms on the exoskeleton, resulting in increased consumption of host tissues (Brown et al. 2012). As molting occurs more frequently in smaller, younger crayfish, their exoskeletons harbor fewer resources for worms which will feed on host tissue when facing resource limitation. Larger cambarid crayfish, which molt infrequently, experience increased epibiotic buildup on their carapace and are more dependent on symbionts for cleaning services (Brown et al. 2012; Skelton et al. 2014; Thomas et al. 2016). This cleaning has been shown to result in increased growth and decreased mortality for medium to large crayfish in the genus *Cambarus* (Brown et al. 2002, 2012; Thomas et al. 2016). Not all crayfish species appear to derive benefits from this symbiosis. The invasive crayfish *F. cristavarius* (Crandall and De Grave 2017) is largely intolerant of these symbionts and readily removes them (Farrell et al. 2014).

While the region has been invaded by a number of crayfish species, our study focused on the effects of *F. cristavarius* on native symbionts. This species is native to West Virginia (Hobbs et al. 1967; Taylor 2000) and was introduced to Mountain Lake, Virginia in the 1930s and likely spread to nearby watersheds via bait dumping by fisherman. Hobbs et al. (1967) suggest that the introduction of *F*. *cristavarius* may have contributed to the decline of at least 2 species of native crayfish in the genus *Cambarus*. This region has also been invaded by the crayfish *F. virilis* and *P. clarkii¸* though neither of these invaders were heavily represented in our sampling locations, and we have considerable background knowledge of *F. cristavarius* from previous research, making it an ideal focal invader.

### Watershed survey

**Fig. 1:**
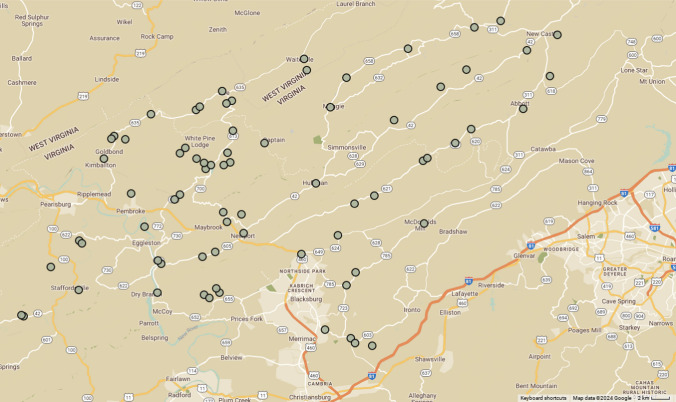
Map of sites from our crayfish and branchiobdellidan survey. The survey included sites in three drainage basins, New River, James, and Roanoke. For spatial reference, the large urban area at the right of the map is the city of Roanoke, VA, and the Virginia-West Virginia border can be seen in the upper left

To investigate potential impacts of crayfish invasion on symbiotic systems under natural conditions, we sampled crayfish at 74 sites from across all three basins in the Mountain Lake region of Virginia (James River Basin: 22 sites, New River Basin: 44 sites, Roanoke River Basin: 8 sites), focusing on smaller streams, covering an area of ≈ 600 km^2^ (Fig. 1). At each site, we sampled crayfish using substrate disruption and dip netting for 1 person-hour and placed each crayfish into an individual Whirlpak bag to prevent symbiont transfer between hosts. After collection, we determined crayfish host size and species using keys developed for local crayfish (Hobbs et al. 1967; Loughman et al. 2013) and recorded sex of the crayfish and total carapace length (TCL) which is a standard measure of crayfish size. For each sampled site, we calculated the proportion of collected crayfish that were invasive to examine impacts of degree of invasion on symbiont diversity. All branchiobdellidans on collected crayfish were identified to species using Hobbs et al. (1967).

To examine the effects of invasion on native symbiont communities, we used a multiple regression model on two response variables: symbiont richness and symbiont abundance. The two predictors were the proportion of sampled crayfish that were invasive, and TCL. TCL was included as a second factor because branchiobdellidan abundances on native *Cambarus* crayfish vary predictably with host size as a generally positive exponential function (Brown and Creed 2004; Thomas et al. 2016). The distribution of residuals in both models was examined; while residuals for the model of richness were satisfactory, we square-root transformed abundance to better meet the assumptions of multiple regression. While the proportion of the community consisting of invasive crayfish (% invasion) was included as a continuous variable in the overall multiple regression model, to provide better graphical illustration in figures, we binned % invasion into 20% intervals. We also calculated the change in the response x host-size relationship with binned % invasion as the change in slopes from simple linear regressions.

### Host relative abundance experiment

To better understand how the displacement of native *Cambarus* crayfish by invasive *F. cristavarius* affects symbiont communities, we simulated the gradual replacement of native crayfish by invasive crayfish in artificial streams. For these studies, we used Frigid Units Living Stream Systems recirculating flow channels with dimensions of 2.13 m × 0.61 m × 0.53 m. These mesocosms had continuous current velocity (≈ 0.1 m/s) and a constant temperature of 18.33 °C.

We simulated changes in host composition by adjusting the ratio of native to invasive crayfish in 5 combinations: 8:0, 6:2, 4:4, 2:6, and 0:8. Eight crayfish per mesocosm gave a host density of 6.15 crayfish/m^2^, a realistic density for many local streams in the region. Each composition was replicated in four individual mesocosms. Native crayfish were represented by the crayfish *C. appalachiensis* (Loughman et al. 2013) while invasive crayfish were represented by *F. cristavarius*. We used only a single sex, and host sizes within a narrow range (within 3 mm TCL) for each individual mesocosm replicate, though sex and mean size varied in unbiased fashion across replicates. We used the branchiobdellidan *Cambarincola ingens* as a representative symbiont. This large worm is the dominant branchiobdellidan in the symbiont community in the region and has previously been shown to strongly affect fitness of native *Cambarus* hosts (Brown et al. 2002, 2012; Skelton et al. 2016; Thomas et al. 2016).

Fifteen worms were placed on a single, randomly chosen native *C. appalachiensis* in each artificial stream. This design was intended to simulate the scenario in which symbionts have the opportunity to disperse from natives to either other natives or to invaders. In mesocosms representing 100% invasion and thus with only *F. cristivarius* present, one individual was chosen at random to be the initial host. Initial symbiont abundance was higher than normally observed in nature for *C. ingens* (≈ 2×) so as to stimulate dispersal away from the initial host and compensate for symbiont mortality, since this branchiobdellidan species has been shown to disperse in response to overcrowding (Skelton et al. 2015). Prior to the initiation of the experiment, existing branchiobdellidan symbionts and their cocoons were removed using a 10% MgCl solution (Brown et al. 2002) and each individual crayfish was marked with colored polymer lacquer to enable us to track the numbers of symbionts on each host. Observations of symbiont abundance and survival per experimental unit were made every day for three days to capture early dispersal from the initial host. Because symbiont dispersal slowed as symbiont abundance was reduced below elevated levels, subsequent observations were made every three days.

We examined the relationships between symbiont dispersal and survival, and the proportion of invasive crayfish in each treatment. On each measurement date, we assessed the number of branchiobdellidans on each host using visual counts under a stereomicroscope. The large size and known preferred locations on hosts of *C. ingens* (Skelton et al. 2015) allowed for accurate visual counts, though occasionally missing an individual was possible. Symbiont dispersal was determined as the proportion of worms that had dispersed away from the original host and onto a new host. Symbiont survival was determined as the proportion of worms that were still surviving at each observation. Data were analyzed using a linear mixed model in a repeated measures analysis to determine if proportion of invasive crayfish affected symbiont dispersal and survival *lme()* function in the R package *nmle* (R Core Team 2016). The proportion of invasive crayfish and days since the initiation of the experiment were included as fixed effects in the model and the experimental unit was included as a random effect to control for the inherent autocorrelation introduced by repeated measures. Model residuals were examined for deviations from normality and heteroscedasticity using residual plots.

## Results

### Surveys

**Fig. 2:**
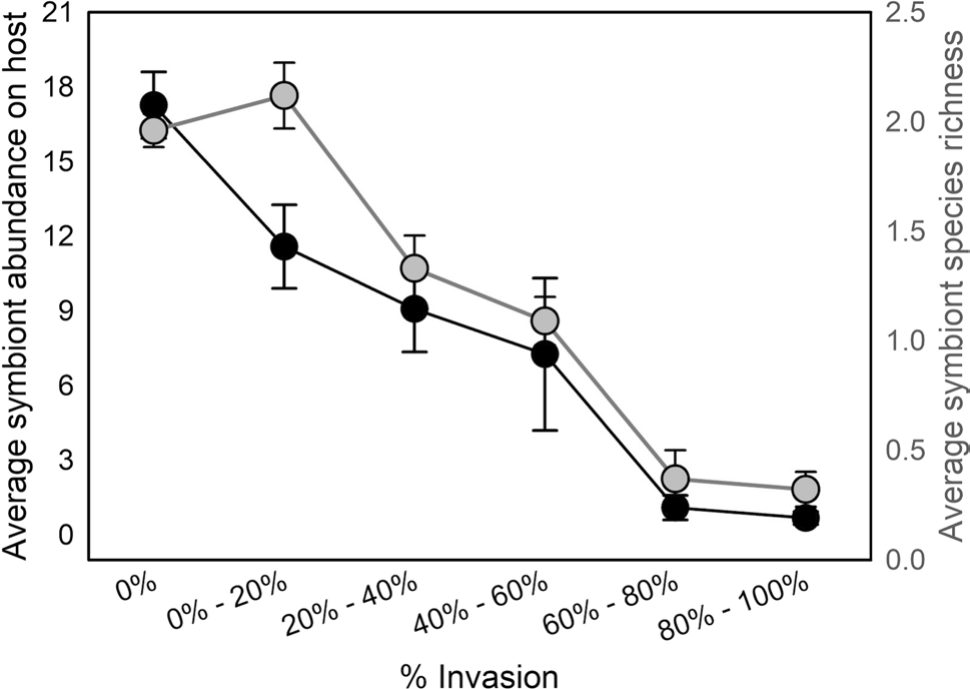
Average symbiont abundance (black points and lines) and richness (grey points and lines) on crayfish hosts collected from sites with no invasive crayfish (0% of collected crayfish) to a high percentage of invasive hosts (80–100% of collected crayfish). Error bars = 1 SE

**Fig. 3:**
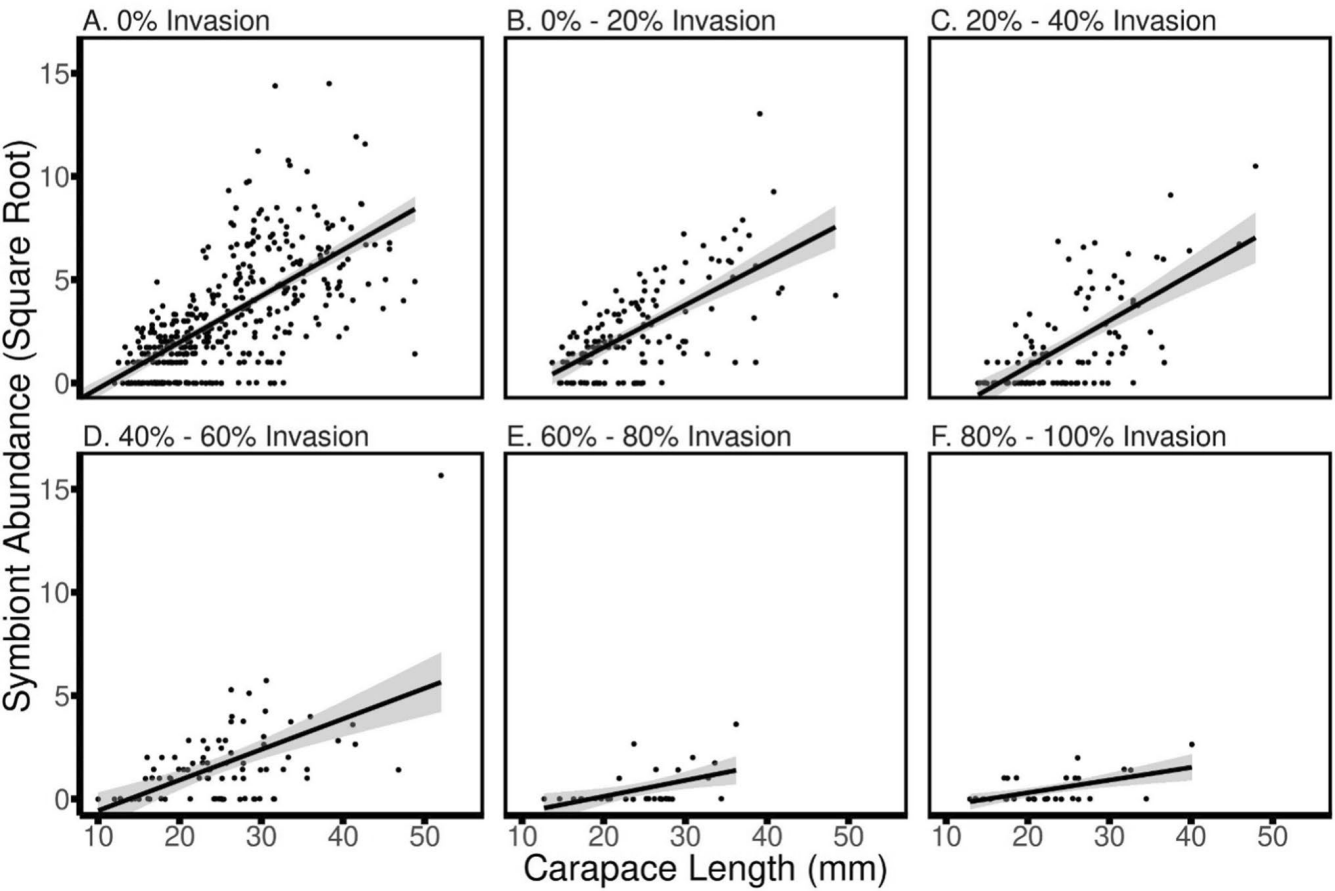
Relationship between the square root of symbiont abundance and carapace length of individual crayfish hosts as sites are increasingly affected by invasion. Each point represents the symbionts community of an individual crayfish of a given size

**Fig. 4:**
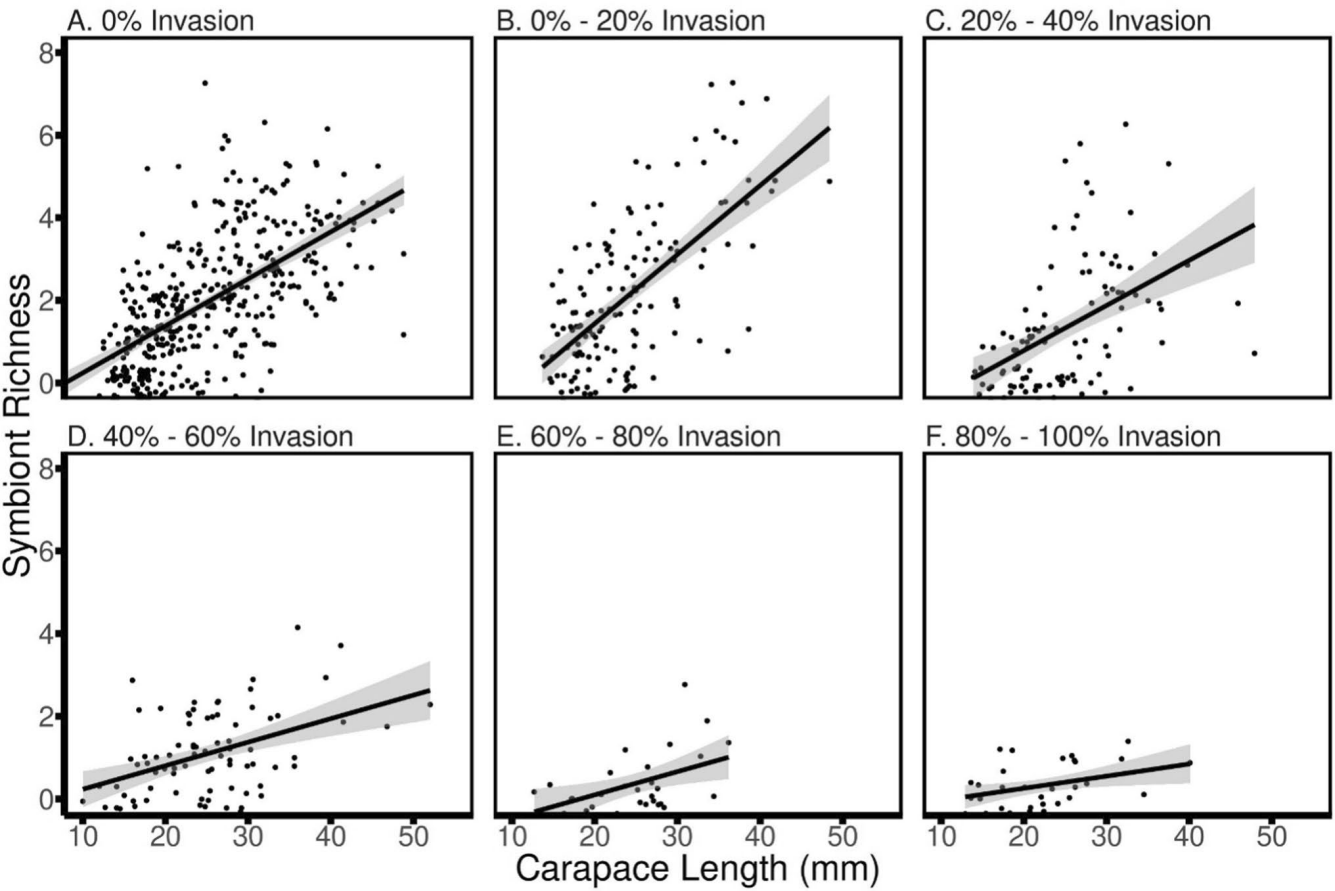
Relationship between the square root of symbiont species richness and carapace length of individual crayfish hosts as sites are increasingly affected by invasion. Each point represents the symbiont community of an individual crayfish of a given size

As the proportion of invasive crayfish at a site increased, there were dramatic decreases in branchiobdellidan abundance and richness. In total, 789 crayfish and 10,137 branchiobdellidans were collected during the survey portion of this study (Table 1). Both symbiont abundance and richness showed near-linear declines across the invasion gradient, with mean abundance decreasing 96% and mean richness decreasing 84% from 0 to 100% invasion (Fig. 2). Multiple regression supported a significant direct effect of % invasion on both symbiont abundance and richness (Table 2). There was no direct effect of host size on either abundance or richness, but there was a significant interaction with crayfish size for both responses (Tables 2 and 3). The reason for a lack of a direct host size effect was apparent when examining the relationship between both abundance and richness with host size across the binned intervals of % invasion: Invasion resulted in a decoupling of both symbiont abundance (Fig. 3) and symbiont richness (Fig. 4) from host size. While the slopes of the abundance/richness × host size relationships were all positive and significant, there was a dramatic decrease in the slope of the relationship for both abundance (from 0.223 to 0.0562) and richness (from 0.114 to 0.0294), decreases in slope of 75% and 74%, respectively (Table 3).

**Table 1:** Total counts of crayfish and branchiobdellidans collected during the regional survey

Crayfish species		Branchiobdellidan species	
Cambarus appalachiensis	275	Bdellodrilus illuminatus	586
Cambarus bartonii	207	Pterodrilus alcicornus	2199
Cambarus longulus	167	Cambarincola fallax	2810
Cambarus accuminatus	4	Cambarincola philadelphica	1425
Faxonius cristavarius	130	Cambarincola holostoma	137
Faxonius virilis	6	Cambarincola heterognatha	351
		Cambarincola ingens	383
		Cambarincola branchiophilia	329
		Ankyrorilus koroneaeus	1640
		Ankyrodrilus legaeus	15
		Xironogiton ilusformosus	2
		Xironogiton instabilius	260

For crayfish, underlined text indicates an invasive species

**Table 2:** Results of multiple regression models of total symbiont abundance and symbiont species richness, from survey data, as a function of % invasion at a site and crayfish host size (total carapace length, TCL)

Parameter	*df*	Sums of squares	*F*	*p*
Total symbiont abundance				
% invasion	1	503.8	101.2	< 0.0001
Carapace length	1	1.2	0.25	0.62
%Inv × TCL	1	795.5	159.7	< 0.0001
Residuals	786			
Symbiont species richness				
% invasion	1	210.9	112.6	< 0.0001
Carapace length	1	0.09	0.05	0.83
%Inv × TCL	1	255.7	136.5	< 0.0001
Residuals	786

**Table 3:** Simple linear regression results for total symbiont abundance and symbiont species richness as a function of host size (carapace length) for each bin of the % invasion gradient from survey data

% invasion	*r*^2^	*p*	Slope
Total symbiont abundance			
0%	0.4501	< 0.0001	0.2237
0–20%	0.4176	< 0.0001	0.2053
20–40%	0.4193	< 0.0001	0.2238
40–60%	0.2822	< 0.0001	0.1479
60–80%	0.2138	0.0058	0.0777
80–100%	0.3001	0.00082	0.0562
Symbiont species richness			
0%	0.375	< 0.0001	0.1137
0–20%	0.434	< 0.0001	0.1671
20–40%	0.2299	< 0.0001	0.1090
40–60%	0.1906	< 0.0001	0.0569
60–80%	0.1885	0.00958	0.0562
80–100%	0.1389	0.0222	0.0294

Data are the same as in Table 2, but analyzed individually in % invasion bins of 20% to correspond with Figs. 2 and 3

### Host relative abundance experiment

**Fig. 5:**
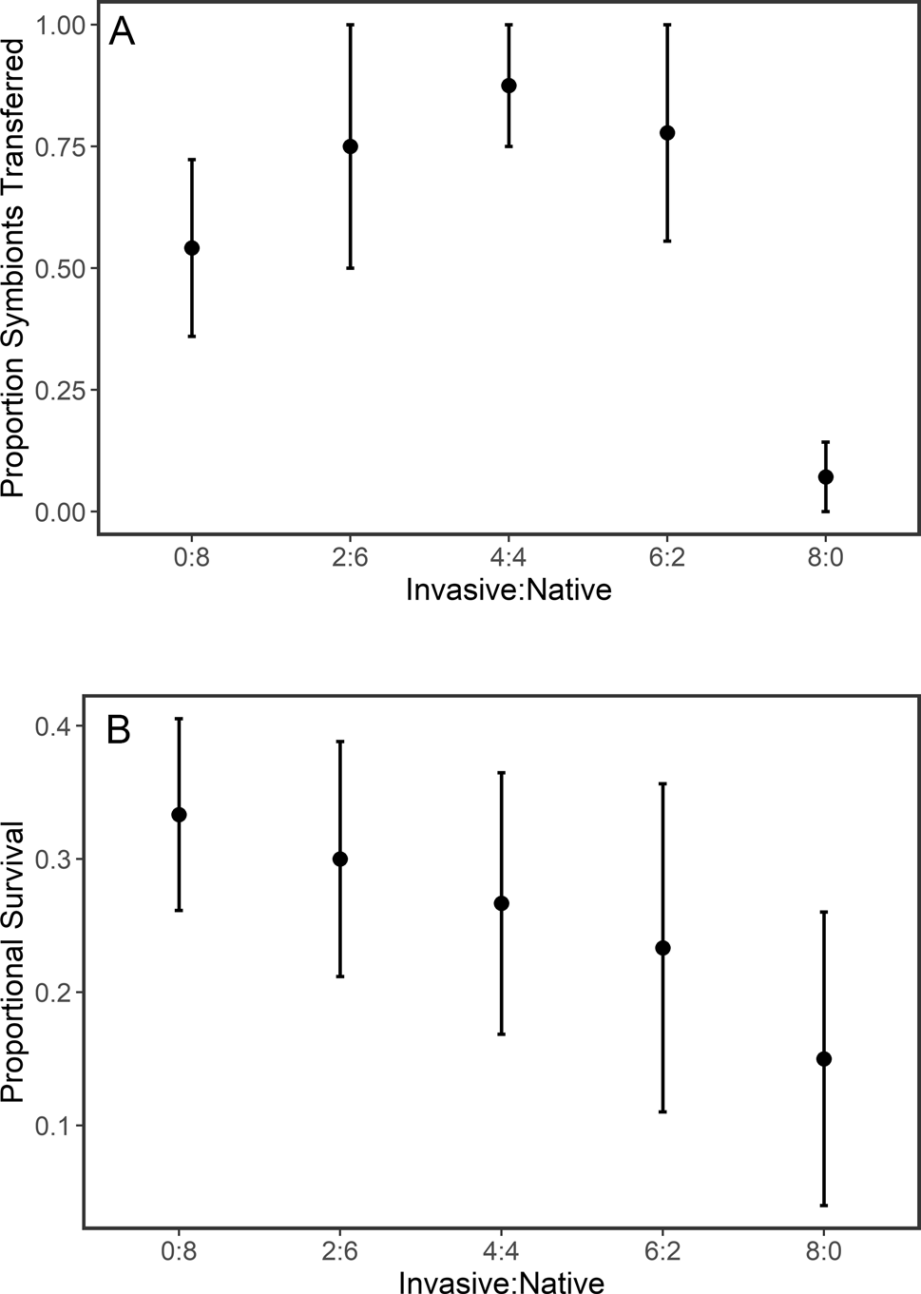
**A** Results of the host replacement experiment. **A** Proportion of symbionts that dispersed from the original host as native crayfish are progressively replaced from a fully native host community (8:0) to a fully invasive host community (0:8). Points represent the proportion of symbionts that had dispersed by the final day of the experiment. Dispersal was calculated as the number of symbionts that had transferred to a new host, as a proportion of the current remaining symbiont count. **B** Proportion of symbionts that survived to the final day of the displacement study as native crayfish are progressively displaced from a fully native host community (0:8) to a fully invasive host community (8:0). Points represent the proportion of symbionts that had survived to the final day of the experiment. Means (± 1 SE) are shown. *N* = 4 for each treatment

Replacement of native host crayfish by invasive crayfish under controlled experimental settings resulted in decreases in both successful worm dispersal among crayfish hosts (Fig. 5A) and in worm survival (Fig. 4B). Replacement of native hosts initially slightly promoted the dispersal of symbionts; however, dispersal decreased precipitously when all native crayfish were replaced (Fig. 4A, time series in Supplementary Fig. 2A), though this lowered dispersal rate may have been as much the result of removal by invasive *Faxonius* as a change in dispersal behavior. Symbiont survival decreased linearly with native host displacement and showed a 57% reduction in survival rate across a gradient from 0% invasives to 100% invasives. Linear models showed that both host composition and time, i.e., days since initiation of the experiment, had strong effects on both worm dispersal and survival (Table 4, time series in Supplementary Fig. 2B).

**Table 4:** Summary of results from linear mixed model examining significance of relationships between both % invasion and days since initiation of experiment on symbiont survival and transfer between hosts

Response variable	% invasion	Days since initiation	Interaction
Symbiont survival	< 0.0001	< 0.0001	0.1448
Symbiont transfer	< 0.0001	0.0166	0.3683

## Discussion

Our results from both field sampling and mesocosm experiments clearly demonstrate that invasion by non-native hosts was correlated with sharp declines in both abundance and diversity of native symbionts. While the positive influence of host size on both symbiont abundance and diversity persisted at all levels of invasion, the slope of that relationship decreased ≈75% for both responses as the relative abundance of the invading host increased. Furthermore, our experiment, combined with knowledge from previous work, strongly suggested the mechanisms behind these declines.

The mechanisms underlying the negative impacts of *Faxonius* crayfish on these symbiont communities appear to be driven by two factors. The first of these is the non-competence of invasive *Faxonius* for native symbionts from this region (Farrell et al. 2014). The crayfish *F. cristavarius* removes ectosymbionts at a rate 4 × higher than that of native hosts (Farrell et al. 2014). It is worth noting that species of *Faxonius* are common hosts to branchiobdellidan symbionts in their native ranges (Gelder et al. 2009; Williams et al. 2009), so the intensity of *Faxonius*’ response to branchiobdellidans in this region may be a product of lack of familiarity between host and symbiont. The second mechanism is imperfect discrimination in host selection when symbionts disperse. In the experiment, branchiobdellidan dispersal increased slightly at intermediate levels of invasive hosts. This initial increase in dispersal was likely due to behavioral differences between native *Cambarus* and invasive *Faxonius* crayfish (Supplemental Fig. S1A & B). *Cambarus* are typically less mobile, spending more time stationary under rocky substrate than *Faxonius* (Anastácio et al. 2015; Hirsch et al. 2016; Loughman et al. 2013). As a result, as the percentage of invasive hosts increased in both the surveyed communities and in the experiment, it is likely that there was also an increase in crayfish encounter rates created by higher natural movement rates of *Faxonius* which also likely encourage native *Cambarus* to move more often because of encounters with exploring *Faxonius*. This increased rate of host encounters may have provided more opportunities for the symbionts to disperse since this species of branchiobdellidan requires contact between hosts for dispersal to occur. However, increased dispersal did not translate into increased survival. With progressive replacement of native hosts, symbionts were increasingly likely to disperse onto the noncompetent, invasive *Faxonius* and not emigrate before being removed. Thus, these results demonstrate that invasive hosts may not only affect symbiont survival on the host, but may also affect symbiont dispersal behavior.

Previous experiments have demonstrated that branchiobdellidans, including the species used in this study, discriminate between hosts, but that discrimination is imperfect with symbionts choosing to disperse to native rather than invasive crayfish by a ratio of ≈ 3:1 (Brown and Creed 2004). Symbionts that disperse to noncompetent invasive hosts may be removed by the host before dispersing back to a competent, native host. This mechanism may explain why symbiont dispersal decreased to nearly zero when total replacement of native hosts occurred in our experiment. When symbionts dispersed across fully invaded host communities, they appear to have been removed too quickly for dispersal success to be recorded, a mechanism that is also supported by the linear decrease in survivorship with increased representation of invasive hosts from survey data. A reason for dispersing from native hosts, despite some ability to discriminate between hosts, is likely because of local limitations in resources such as food or prime reproductive sites (Creed and Brown 2018; Skelton et al. 2015; Thomas et al. 2013). In a relevant prior study, Brown and Creed (2004) performed a 24-h host choice experiment in which branchiobdellidans preferentially colonized native hosts at a 3:1 ratio with symbiont loss rates ≈ 20%. In the current experiment, there were higher symbiont loss rates of up to 60% per 24 h in the 100% invasion treatment group. These increased loss rates were likely a result of a larger experimental environment that increased the likelihood of worm removal by *Faxonius* before they encountered a *Cambarus* host. The current experiment used 8 crayfish in Living Stream mesocosms of 530 L vs. the 1 L microcosm of Brown and Creed (2004). Additionally, in the experiment of Brown and Creed (2004), branchiobdellidans were placed on a centralized, inanimate substrate rather than a living host, and thus were alleviated from the immediate pressures of host control mechanisms and allowed to choose a host at their leisure. Therefore, while the loss rates observed in our experiment were high relative to previous studies, they were likely more representative of branchiobdellidans dispersing in field conditions.

We focus on host invasion as the primary variable responsible for the decreases in symbiont diversity and abundance observed in our survey. However, it is possible that other variables like water quality, land-use, and in-stream habitat might have had an influence as well. While we did not collect data on these variables, we appeal to two aspects of our survey and its results to address this issue. The first is the extent of our survey (≈ 600 km^2^) and our strong coverage within this area. The extent of the survey is important because it captured a range of possible covariates, and despite not including them in our analysis, we still see the strong signal of % invasion. A second point is that sites that were close to each other, and likely environmentally similar in most respects, had very different levels of symbiont abundance and diversity depending on level of invasion. For example, several of our sites in the James basin were in close proximity to one another, but 5 sites were uninvaded, while 3 sites had invasion above 60%. In uninvaded sites, average cumulative symbiont diversity for a site was 2.48 (Shannon index) and average abundance was 36 symbionts. In contrast, in the invaded sites, diversity was 1.33 and average abundance was 4.33. We do not suggest that environmental covariates are not potentially important. In fact, *F. cristavarius*’ ability to use sediment as a resource likely contributes to invasions in more highly sedimented areas (Helms and Creed 2005). Regardless, the signal of invasion is strong, despite potential residual effects of these potential covariates. An additional alternative hypothesis is that invaded sites simply have lower host densities that can provide habitat for branchiobdellidans. However, when we examined compiled surveys from the region, we found no evidence of a relationship between total crayfish abundance and % invasion (slope = 0.012, *r*^2^ = 0.005; Supplemental Fig. 1).

While our focus in this study has largely been on the effects of invasion on symbionts, the consequences for hosts may be no less dramatic. Previous research showed that a number of crayfish species from multiple geographic regions are engaged in a mutualism with branchiobdellidans (Brown et al. 2002, 2012; Lee et al. 2009; Ames et al. 2015; Thomas et al. 2016). There are likely many more crayfish species engaged in mutualistic interactions with branchiobdellidans than are currently described, since the majority of studies examining this interaction have described mutualistic effects. Previous work has found that the mutualism can significantly decrease host mortality and increase host growth rates by up to 45% compared to symbiont-free controls (Brown et al. 2002, 2012). The presumed mechanism underlying this mutualism is removal of fouling material (bacteria, detritus) from the gills, which improves gas exchange and ammonia excretion (Brown et al. 2002, 2012, Creed and Brown, unpublished data). When branchiobdellidans are food limited on their hosts, they may switch from cleaning accumulated bacteria and detritus found on the host’s gills, to consuming this gill tissue, effectively switching from mutualists to parasites (Brown et al. 2012).

As noncompetent invasive hosts increasingly displaced more competent native hosts, there was a decoupling of the relationship between host size and both symbiont abundance and diversity. Larger crayfish collected at sites dominated by native *Cambarus* crayfish hosted more branchiobdellidans as well as a greater diversity of these worms. In addition to simply providing more surface area for colonization, larger *Cambarus* crayfish exhibit reduced symbiont control behaviors as well as decreased molting periodicity (Skelton et al. 2014, 2015; Thomas et al. 2016). It is these larger crayfish that are the beneficiaries of this mutualism. Slopes of the relationships between host size and both symbiont abundance and richness were drastically reduced in sites dominated by non-native hosts. Reductions in symbiont abundance and diversity, especially on larger native crayfish, appeared to reflect that as invasive hosts displaced native hosts, the integrity of the native symbiotic system was degraded.

Invasions can have negative effects on native symbiont abundance and diversity as the proportion of native hosts declines in a community and if the invaders are unsuitable or non-competent hosts (Civitello et al. 2015; Huang et al. 2016; Johnson et al. 2013; Keesing et al. 2006; Let et al. 2023). This phenomenon is known as the dilution effect and was initially used to describe the effect that varying host competence had on the abundance of parasitic symbionts in or on their target hosts. With increasing host diversity, parasites are more likely to encounter non-competent hosts when they disperse, with the result that parasite prevalence in suitable, target hosts should be lower in a more diverse host community (Civitello et al. 2015). This reduction in parasite prevalence was viewed as a positive effect of higher host diversity (Civitello et al. 2015; Huang et al. 2016). Our research demonstrates that the reverse can be the case in systems of mutualistic and commensalistic symbionts. Previous studies have also described the effect of “mutualism disruption” in which invasive species decouple the links between hosts and mutualistic symbionts and therefore indirectly facilitate invasion (Roche et al. 2023). While we have no direct evidence that mutualism disruption is facilitating invasions in this region, such an effect would be highly likely given that branchiobellidan symbionts have been experimentally demonstrated to increase the growth of native hosts by up to 45% (Brown et al. 2012).

When invasions occur, native symbiont communities are often overlooked. They may be directly affected by invasive hosts, and in the case of mutualists that positively affect native hosts, the loss of mutualistic effects for natives may contribute to the potential success of invaders (Creed et al. 2022b; Richardson et al. 2000a; Traveset and Richardson 2010, 2014). This oversight is especially true for invasions involving animal hosts; more information has been accrued for invading plants and their interactions with native symbionts (Richardson et al. 2000a, b; Traveset and Richardson 2014). Our results show that the spread of invasive crayfish in three different watersheds in the Mountain Lake Region of Virginia is having negative impacts on native symbiont communities in the form of decreased species richness and abundances of symbionts. As some of these branchiobdellidans are engaged in mutualisms with their native crayfish hosts, fitness of native hosts will decline with the loss of these mutualists (Brown et al. 2002, 2012; Thomas et al. 2016). The fitness reductions associated with the loss of mutualists may further exacerbate the loss of native hosts. These hidden effects of invasion may be occurring in many different invasion scenarios and contributing to the decline or even extinction of native host and symbiont species around the world. We are hardly the first to recognize the importance of incorporating symbionts into conservation and management plans (e.g., Aslan et al. 2013; Brodie et al. 2014; Carlson et al. 2020; Let et al. 2023), but we add our voices to this growing call.

### Supplementary Information

Below is the link to the electronic supplementary material.

Supplementary file1 (DOCX 311 KB)

## Data Availability

Data from this publication will be archived at the Virginia Tech Libraries Data Archive following publication. https://guides.lib.vt.edu/VirginiaTechDataRepository.
