# Supplementary material for: Impacts of invasion on a freshwater cleaning symbiosis
Source: Oecologia. 2024 Aug 3;205(3-4):669–80. doi: 10.1007/s00442-024-05600-4 (PMC11358191; doi:10.1007/s00442-024-05600-4)

Impacts of Invasion on a Freshwater Cleaning Symbiosis: SUPPLEMENTAL MATERIAL

Spencer S. Bell^1^

Philip McElmurray^1,3^

Robert P. Creed^2^

Bryan L. Brown^1,4^

1 - Dept. of Biological Sciences, Virginia Tech, Blacksburg, VA 24061 USA

2 - Dept. of Biology, Appalachian State University, Boone, NC 28608

3 - Current address: Dept. of Anthropology, Washington University in St. Louis, St. Louis, MO

63130 USA

4 – Address correspondence to Bryan L. Brown, Department of Biological Sciences, Virginia Tech; [stonefly@vt.edu](mailto:stonefly@vt.edu)

**Supplementary Figures**

Figure S1: Compiled data from multiple surveys in the study region examining the relationship between crayfish abundance and level of invadedness at each site. Each point represents a site sampled quantitatively and the dotted line shows the least-squares regression line for the data (slope = 0.012, r^2^ = 0.005).


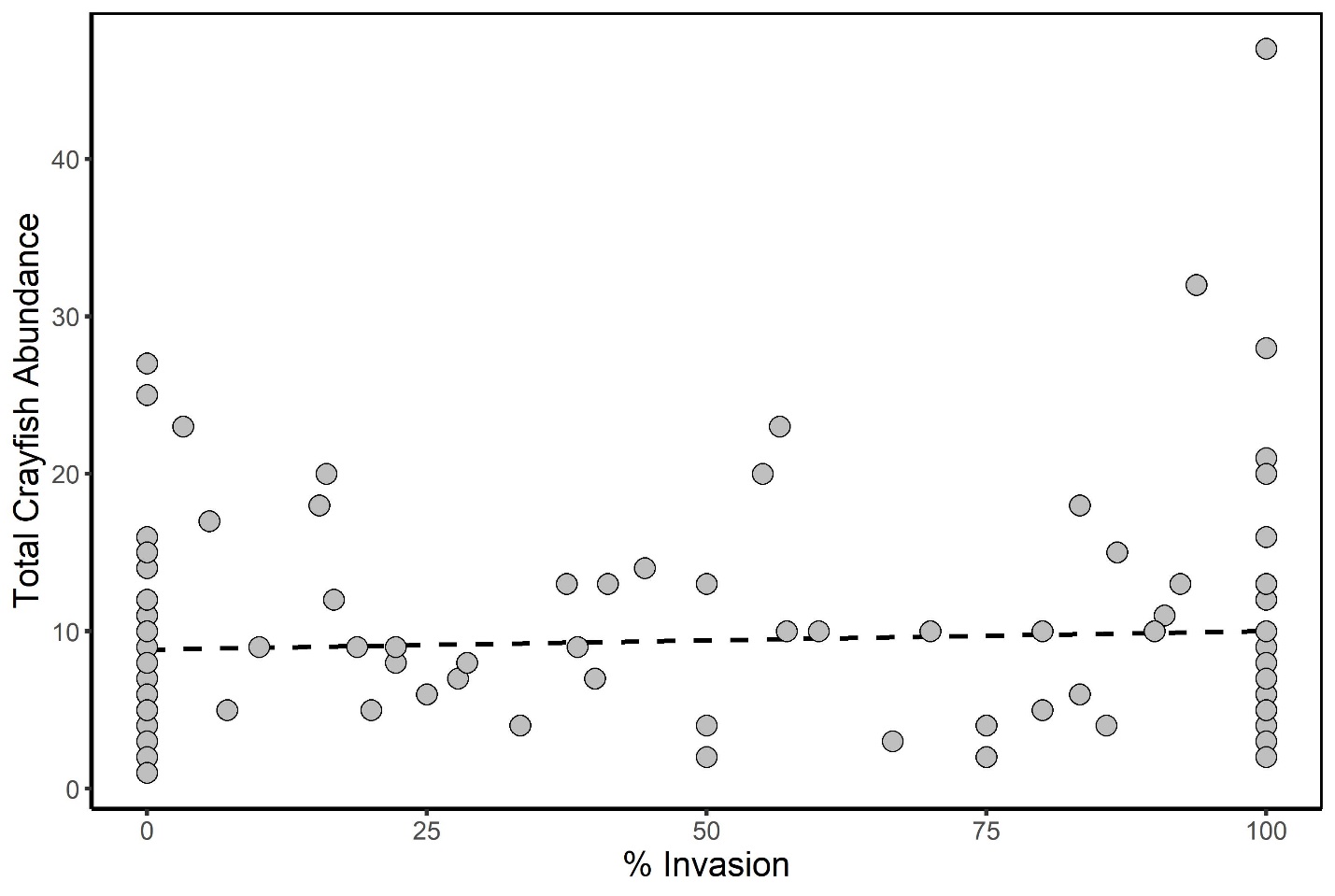


Figure S2a: Time series of results from the Host Relative Abundance Experiment showing % symbionts transferred on each sampling day of the experiment. Results in Figure 4 are from the same experiment but only show the day 16 results. Points are means ± 1SE


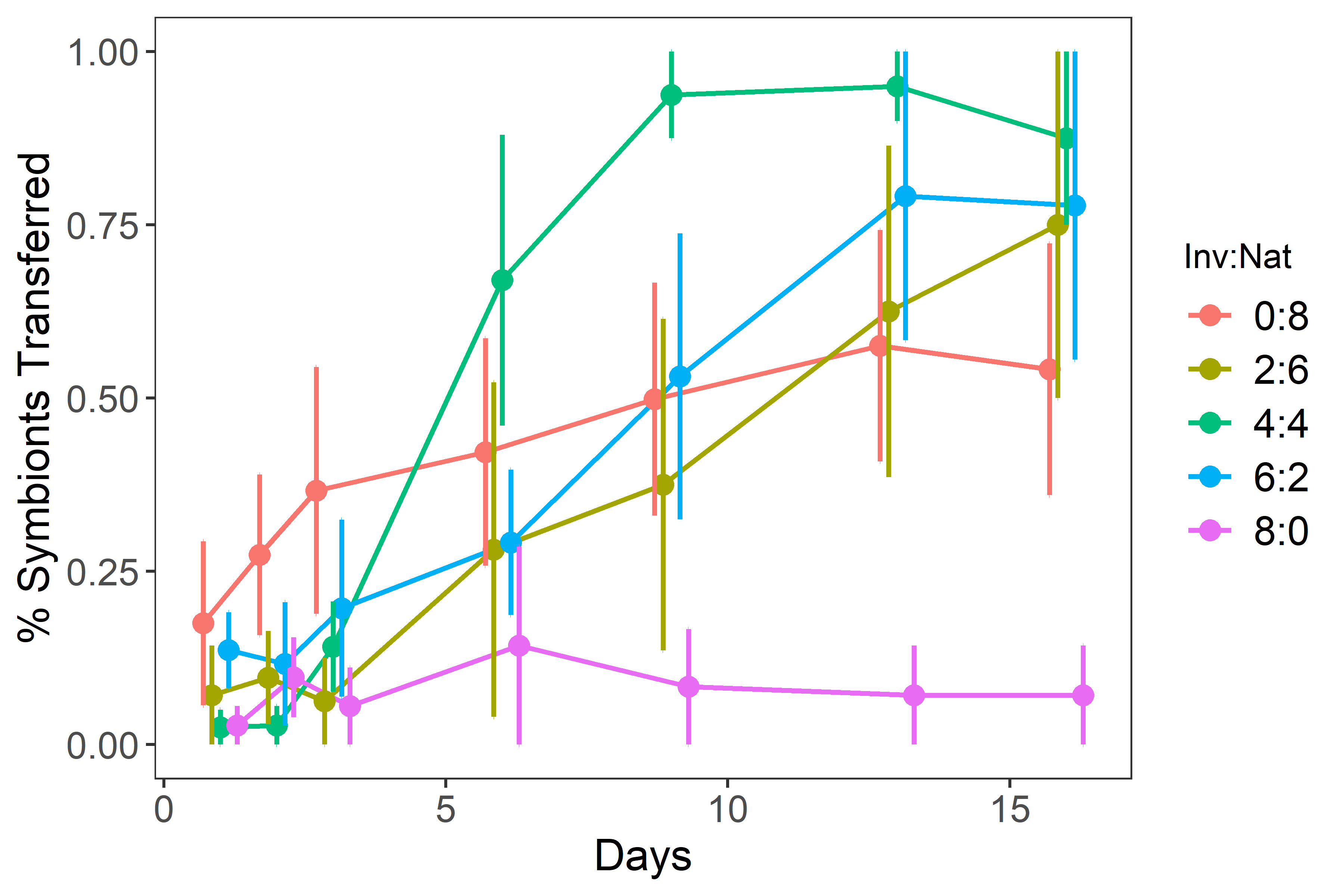


Figure S2b: Time series of results from the Host Relative Abundance Experiment showing % survival of symbionts on each sampling day of the experiment. Results in Figure 4 are from the same experiment but only show the day 16 results. Points are means ± 1SE


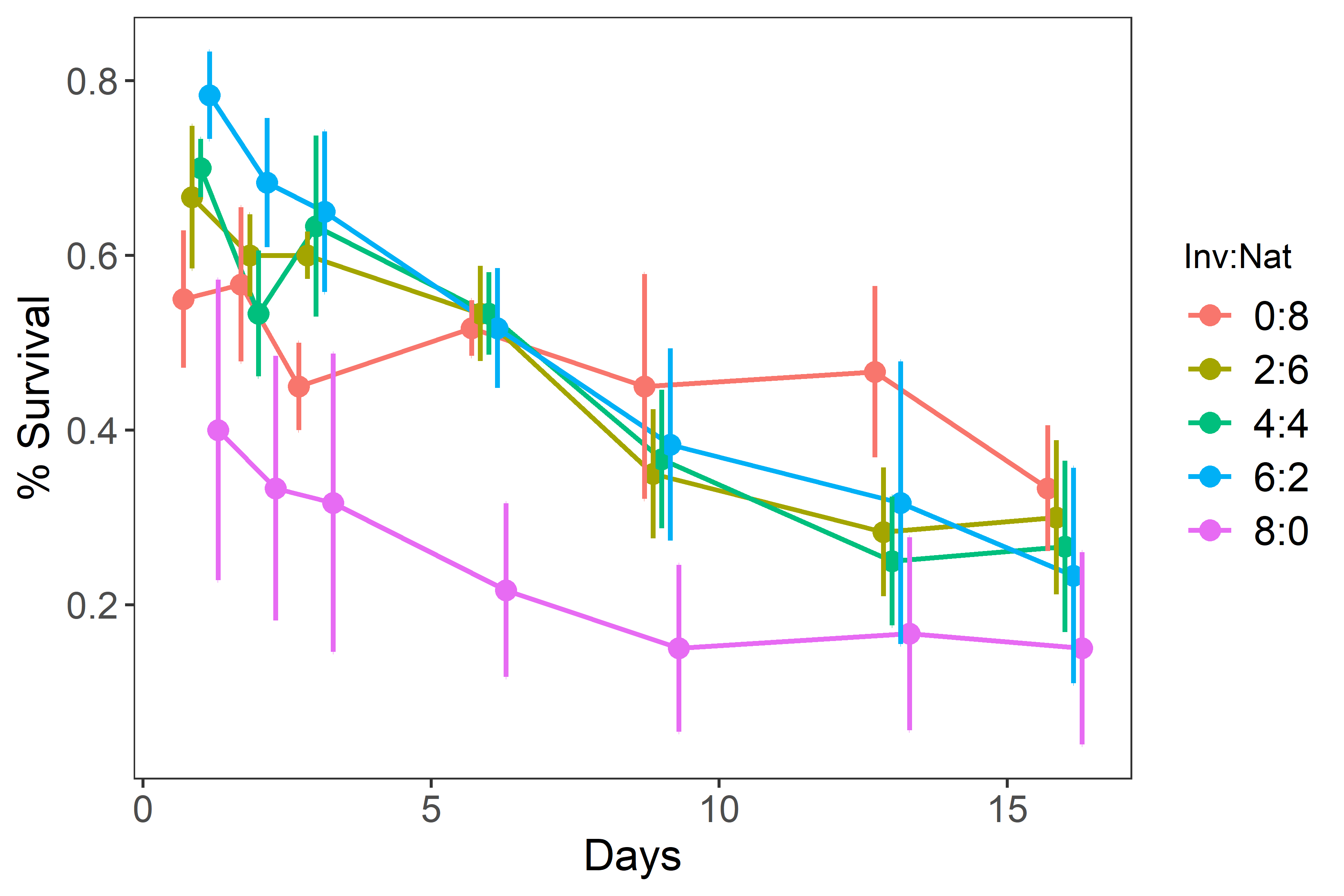

Supplement: Supplementary file 1 — Supplementary file1 (DOCX 311 KB) [file 442_2024_5600_MOESM1_ESM.docx]
